# Detection, characterization and regulation of antisense transcripts in HIV-1

**DOI:** 10.1186/1742-4690-4-71

**Published:** 2007-10-02

**Authors:** Sébastien Landry, Marilène Halin, Sylvain Lefort, Brigitte Audet, Catherine Vaquero, Jean-Michel Mesnard, Benoit Barbeau

**Affiliations:** 1 Université du Québec à Montréal, Département des sciences biologiques, Montréal (Québec), H2X 3X8, Canada; 2 Centre de Recherche en Infectiologie, Centre Hospitalier Universitaire de Québec, Pavillon CHUL, and Département de Biologie médicale, Faculté de Médecine, Université Laval, Ste-Foy (Québec), G1V 4G2, Canada; 3 INSERM U511, UPMC-Paris VI, Pitié-Salpêtrière, Paris, France; 4 Laboratoire Infections Rétrovirales et Signalisation cellulaire, CNRS/UM I UMR 5121/IFR 122, Institut de Biologie, 34960 Cedex 2, Montpellier, France

## Abstract

**Background:**

We and others have recently demonstrated that the human retrovirus HTLV-I was producing a spliced antisense transcript, which led to the synthesis of the HBZ protein. The objective of the present study was to demonstrate the existence of antisense transcription in HIV-1 and to provide a better characterization of the transcript and its regulation.

**Results:**

Initial experiments conducted by standard RT-PCR analysis in latently infected J1.1 cell line and pNL4.3-transfected 293T cells confirmed the existence of antisense transcription in HIV-1. A more adapted RT-PCR protocol with limited RT-PCR artefacts also led to a successful detection of antisense transcripts in several infected cell lines. RACE analyses demonstrated the existence of several transcription initiation sites mapping near the 5' border of the 3'LTR (in the antisense strand). Interestingly, a new polyA signal was identified on the antisense strand and harboured the polyA signal consensus sequence. Transfection experiments in 293T and Jurkat cells with an antisense luciferase-expressing NL4.3 proviral DNA showed luciferase reporter gene expression, which was further induced by various T-cell activators. In addition, the viral Tat protein was found to be a positive modulator of antisense transcription by transient and stable transfections of this proviral DNA construct. RT-PCR analyses in 293T cells stably transfected with a pNL4.3-derived construct further confirmed these results. Infection of 293T, Jurkat, SupT1, U937 and CEMT4 cells with pseudotyped virions produced from the antisense luciferase-expressing NL4.3 DNA clone led to the production of an AZT-sensitive luciferase signal, which was however less pronounced than the signal from NL4.3Luc-infected cells.

**Conclusion:**

These results demonstrate for the first time that antisense transcription exists in HIV-1 in the context of infection. Possible translation of the predicted antisense ORF in this transcript should thus be re-examined.

## Background

It has been largely accepted that gene expression in retroviruses solely relies on a single transcript, which is in turn either left unspliced, singly or multiply spliced. This transcript is initiated from the 5' LTR region, which harbours in its U3 segment most of the necessary binding sites for important transcription factors regulating the expression of retroviral genes. In addition, for all studied retroviruses, this transcript initiates at a single position and is typically dependent on an upstream TATA box. Few studies have addressed the possible existence of transcripts initiated at other position in the retroviral genome. A number of reports have however provided an interesting and unexpected possibility to retroviral gene expression. Indeed, in a few complex retroviruses including HIV-1, FIV-1 and HTLV-I, it has been suggested that transcripts produced in the antisense direction exist and that these transcripts could have the potential to encode for a protein [1-4]. Although these results have been debated and largely contested, new results obtained with the HTLV-I virus have importantly revived the issue over antisense transcription [2,5-15]. Indeed, the HTLV-I retrovirus has been the first retrovirus from which the existence of an antisense transcript has been clearly demonstrated. Recent studies have further highlighted the spliced nature of this transcript [12-14]. The HBZ protein encoded from this transcript was shown to have AP-1 and Tax inhibitory activity and to be detected in infected cell lines as well as PBMCs from HTLV-I infected individuals.

The existence of antisense transcription in HIV-1 has been similarly suggested based on the identification of a conserved ORF in the antisense strand of its genome. Hence an initial study by Miller had identified a well conserved ORF of 189 amino acids, later termed ASP (Antisense Protein) on the antisense strand, which was generally well conserved in all analysed HIV-1 proviral DNA [3]. Analysis of the hydrophobic profile of the potentially encoded protein revealed it to be highly hydrophobic and thus to possibly be associated to the membrane. Detection of the ASP protein has only been possible through Western blot analysis of bacterially produced ASP and electron microscopy studies [16]. Despite these studies, no functions have yet been assigned to this potential virally encoded protein and its existence remains controversial.

Studies have however been more focussed on the detection of the antisense transcript itself in HIV-1. The existence of the transcript has been previously suggested through Northern blot and RT-PCR approaches [4,17,18]. Studies based on the identification of the 5' and 3' ends of the antisense transcript have also been performed and suggested that this transcript was initiating next to the 3' LTR border, although no consensus was obtained [4,19,20]. Promoter analyses have been further conducted by using the isolated 3' LTR positioned in the antisense orientation and T-cell activators were shown to positively modulate promoter activity while Tat had an adverse effect [4,19,21]. Although these analyses have tended to infer that this pattern of expression was occurring in HIV-1, numerous artefacts and contradictory results have not permitted to unequivocally demonstrate that indeed HIV-1 antisense transcription existed. Therefore reassessment of antisense expression is directly needed to readdress the existence of antisense transcription in HIV-1.

In virtue of the recent results on HTLV-I antisense transcription, the goal of this study was to readdress the existence of antisense transcription in HIV-1. Using an antisense transcription-specific RT-PCR approach (with no non-specific RT priming artefact) and an HIV-1 proviral DNA construct expressing the luciferase gene in the inverse orientation, we provided for the first time strong evidence demonstrating the presence of HIV-1 antisense transcripts. Our data also highlight the existence of a new polyA signal in the antisense strand and strongly support a positive role for Tat on antisense transcription. These results add new important information, which will likely impact on the understanding of HIV-1 replication.

## Results

### Detection of the antisense transcript in infected and transfected cells

**Figure 1:**
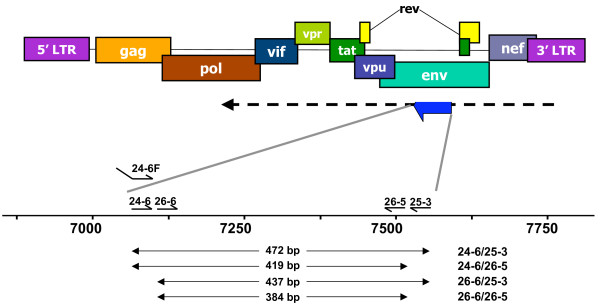
Positioning of the ASP antisense ORF in the HIV-I proviral DNA. The ASP ORF is located on the antisense strand in the region of the *env *gene. Primers used for RT-PCR experiments and the expected sizes of the amplified signals are indicated below the enlarged ASP ORF. The arrow indicated the antisense transcript.

Previous studies had earlier suggested that antisense transcription could be detected through RT-PCR analyses [4]. However, in our hands, these protocols were not suitable for specific detection of antisense transcription as substantial amount of non-specific signals due to endogenous RT priming was apparent. Endogenous RT priming results from priming of RNA by small degraded RNA or DNA fragments present in the extracted RNA pool, which act as primers during the reverse transcriptase step. Given that sense expression in retroviruses has been suggested to be more prominent than antisense transcription, a PCR signal might thus be overwhelmingly derived from cDNA produced from sense mRNA primed by degraded DNA/RNA and not permit to specifically assess the existence of antisense transcripts. Endogenous RT priming is typically controlled by conducting PCR amplification of cDNA produced from RNA in the presence of the reverse transcriptase but in the absence of the RT primer. To detect the antisense transcript, we chose RT and PCR primers in the proviral DNA region located in the ASP ORF. As presented in Figure 1, the ASP ORF is located in the antisense strand in the *env *gene. Several primers were designed to provide signals of different sizes.

**Figure 2:**
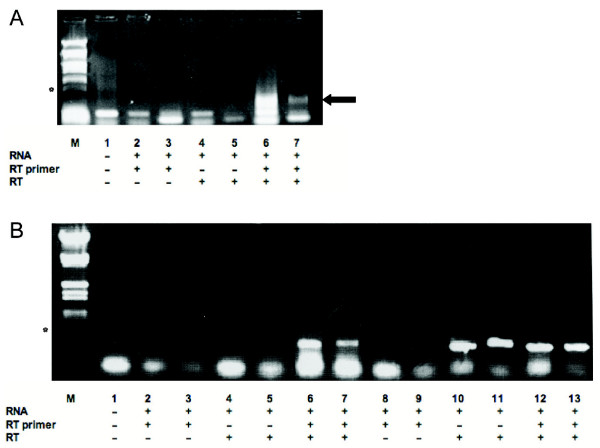
Specific detection of the antisense transcript in latently infected cells and transfected 293T cells (***A***) RT-PCR analyses were performed on RNA samples from J1.1 cells using the 24-6 RT primer and PCR primer combinations 26-6/26-5 (lanes 1,2,4,6) and 26-6/25-3 (lanes 3,5,7) (expected size of 384 bp and 437 bp, respectively). Samples were tested for DNA contamination (lanes 2–3; no RT and no RT primer) and endogenous RT priming (lanes 4–5; RT with no added RT primer). Lane 1 represents PCR analysis with no added cDNA or RNA. Lanes 6 and 7 show the results of PCR using 2 primers combinations. (***B***) 293T cells were transfected with 5 μg of pNL4.3 or pNL4.3ΔNar1 proviral DNA. RT-PCR analysis of RNA samples from transfected 293 T cells and controls were performed as in ***A***. M = Lambda EcoRI/HindIII DNA marker (the asterisk indicates the 564 bp band). The arrows on the right side of panel ***A ***points to the specific signal.

Our first RT-PCR analysis was conducted using standard conditions. However, as indicated above, we expected that most of endogenous RT priming artefacts in HIV-1-infected cells might be coming from cDNA synthesis from the sense transcript occurring through the presence of degraded HIV-1 cellular DNA or antisense RNA. To decrease this important source of endogenous RT priming artefact, we first used the J1.1 cell line, which is latently infected and produces very low amounts of virions when left unstimulated. RNA extracted from this cell line was used for cDNA synthesis with an antisense RNA-specific primer located in the ASP ORF region and PCR was then conducted with two different sets of ASP ORF-derived primers. As presented in Figure 2A, specific signals representing antisense transcription were detected (lanes 6 and 7). The sequencing of these signals confirmed their specificity. Importantly, controls for endogenous RT priming (absence of primer at the RT step) (lanes 4 and 5) and for DNA contamination (no RT step) (lanes 1 to 3) were devoid of any signal further demonstrating that our PCR-amplified fragments were specific to the antisense transcript. Antisense transcription was next tested in 293T cells transfected with HIV-1 proviral DNA. The NL4.3 proviral was thus chosen and, as argued above, a 5' LTR-deleted version termed pNL4.3ΔNarI was generated to minimize endogenous RT priming on sense transcripts. Wild-type and 5' LTR-deleted pNL4.3 constructs were thus transfected in 293T cells and RT-PCR analyses (as described above) were undertaken on RNA samples from these cells (Figure 2B). Again, a specific signal was easily detected in PCR amplification of cDNA originating from 293T cells transfected with pNL4.3ΔNarI (lanes 6 and 7) and its specificity was further demonstrated by sequencing. Controls for DNA contamination or endogenous RT priming indicated no contaminating signals (lanes 1 to 5). As opposed to these results, pNL4.3wt-transfected cells clearly demonstrated the presence of endogenous RT priming, which had a comparable intensity to the signal obtained in the presence of the RT primer (compare lanes 10 and 11 versus 12 and 13 respectively).

**Figure 3:**
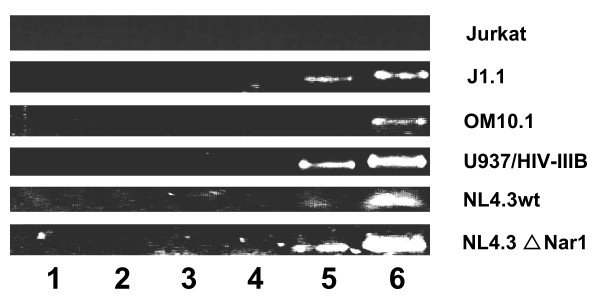
Specific detection of HIV-1 antisense transcript in infected cells lines and transfected 293T cells. Synthesis of cDNA was performed on polyA+ RNA using 24-6 (control RT primer) or 24-6F (floating RT primer) and purified on a PCR-cleanup column. PCR amplifications were performed using the reverse primer 30-20 (anchor) and forward primers 26-5 (lane 1, 3, 5) or 25-3 (lane 2,4 and 6) in order to specifically amplify 24-6F-synthesized cDNA. Samples were tested for anchor primer specificity (lanes 1–2) and cDNA cleanup efficiency (lanes 3–4). Lane 5 and 6 show specific amplification of HIV-1 antisense transcript from J1.1 OM10.1, U937 and ACH-2 chronically infected cells lines and 293T transfected with complete pNL4.3 proviral DNA or 5'LTR-deleted pNL4ΔNar1 construct.

We were next interested in demonstrating the presence of the antisense transcript in chronically infected cells. As we have demonstrated that sense transcription would likely be an important source of endogenous RT priming artefact masking the antisense RNA-specific signal, we thus optimized RT-PCR conditions, which would greatly diminish non-specific signals (see Methods). Tested infected cells included OM10.1, ACH-2, J1.1 and U937 HIV-1_IIIB _(Figure 3 and data not shown). Although the three first cell lines produces low amounts of infectious particles, the U937-infected cell line is known to be a source of substantial levels of produced infectious particles. The improved RT-PCR protocol consisted of an extraction of mRNA followed by an RT step with a primer containing a 3'end complementary to the antisense transcript in the ASP region and a non-complementary 5' end. To remove the RT primer from the reaction, cDNAs are then purified through the use of a column (DNA cleanup step). PCR is then performed with a forward primer again derived from the ASP region and a reverse primer termed the anchor primer specific to the 5' extremity of the RT primer. This RT-PCR approach hence strongly favoured PCR amplification of RT primer-derived cDNAs. Using this approach, we first tested RNA samples from 293T cells transfected with either pNL4.3wt or pNL4.3ΔNarI. As depicted in Figure 3, this approach permitted the detection of the antisense transcript in both transfected cell lines using two different primer sets (lanes 5 and 6). In these experiments, we have also controlled for the specificity of the forward primer used in PCR amplification (primer 30-20 specific to the non-complementary end of the 24-6F RT primer) by testing cDNAs produced with added 24-6 RT primer devoid of the targeted sequence of this forward primer (lanes 1 and 2). Another crucial control consisted of ensuring that no 24-6F RT primer remained in the cDNA reaction after column purification. Sufficient amount of the RT primer during PCR amplification might allow subsequent amplification with the PCR forward primer 30-20 thereby amplifying any source of HIV-1 DNA. Hence, purified cDNA prepared from the RT primer 24-6 was incubated in the presence of an aliquot of mock prepared cDNA from non-transfected 293T cells after column purification (lanes 3 and 4). As expected, none of these controls led to a PCR-amplified signal. With this approach, we next tested the infected cell lines listed above. We indeed demonstrate that this protocol allowed us to specifically detect the antisense transcript in these infected cell lines (lanes 5 and 6) while no undesirable contaminating signals were detected (lanes 1 to 4). None of the RNA samples tested through this protocol had contaminating DNA (data not shown).

These results hence demonstrated the existence of an antisense transcript in HIV-1, which included the ASP ORF sequence. The use of HIV-1 proviral DNA clones and of infected cell lines suggested that a wide range of HIV-1 strains are capable of producing this transcript.

### Identification of multiple transcription initiation sites for the HIV-1 antisense transcript

**Figure 4:**
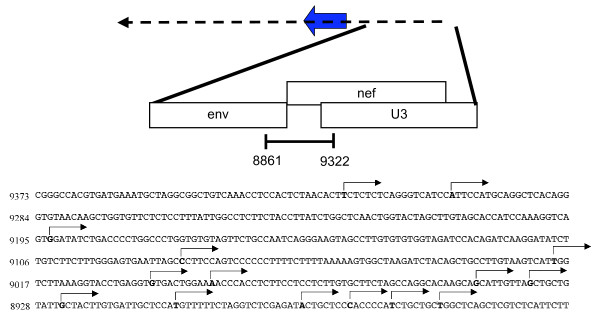
Identification of transcription initiation sites for the antisense transcript. Total RNA from 293T cells transfected with the pNL4ΔNar1 construct was used to amplify 5' cDNA ends using the 5' RACE approach. Numerous transcription initiation sites were identified upon sequencing of multiple PCR-amplified signals and are part of a region presented in the enlarged segment encompassing the LTR, and both *nef *and *env *gene regions. Nucleotide numbering corresponds to the sense strand.

Identification of transcription initiation sites of the antisense transcript of HIV-1 was next undertaken. As an important and specific signal was detected by RT-PCR in 293T cells transfected with pNL4.3ΔNarI, these cells were used as the source of RNA to conduct 5'RACE analyses. In these analyses, PCR amplification was conducted with reverse primers positioned near the 5' end of the ASP ORF region and primers specific to the oligonucleotide ligated to the 5' end of RNAs (as described in Methods). Migration of the 5'RACE products initially indicated the presence of potential multiple transcription initiation sites. Cloning and sequencing of all amplified products generated by 5' RACE (Figure 4) indeed confirmed that numerous transcription initiation sites were identifiable for the HIV-1 antisense transcript and were positioned near the 5'border of the 3' LTR and at more downstream region (in the antisense strand). The majority of the transcription initiation sites were located in a 462 bp region encompassing the transcription initiation site previously described by Peeters *et al*. (1996) [19]. Interestingly, the 3' part of the 462 bp region (containing the previously identified CAP site) presented numerous transcription initiation site identified repeatedly by numerous sequenced PCR amplicons,

These results hence demonstrated that the HIV-1 antisense transcript initiated next to the 3' LTR at multiple positions (in a region ranging in length from 700 to 1250 bp). This multiplicity of initiation sites might be a consequence of the absence of a TATA box at close distance.

### Identification of a new polyA signal in the antisense strand

**Figure 5:**
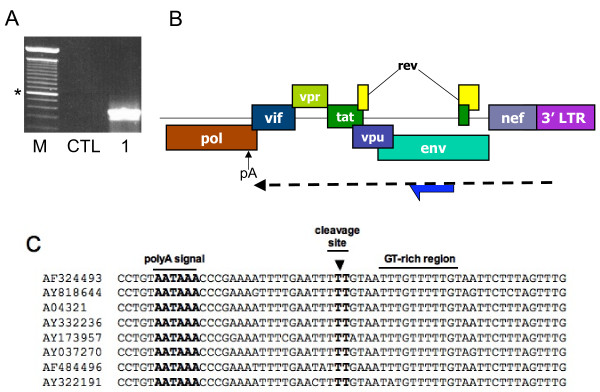
Identification of a new functional polyadenylation site for HIV-1 antisense transcript. (***A***) Total RNA from 293T cells transfected with pNL4.3ΔNarI was used for 3'RACE analysis. CTL represents PCR amplification conducted in the absence of cDNA and RNA samples. The amplified product was cloned and sequenced. M = 100 bp marker (the asterisk indicates the 600 bp band). (***B***) Position of the newly identified antisense polyA addition site (pA indicated with arrow) in the HIV-1 genome. (***C***) Sequence alignment of 8 HIV-1 genome showing conservation for the consensus polyA signal and of a GT-rich consensus sequence. Position of the polyA addition site is indicated by an arrow.

Previous results had suggested that non-consensus polyA signals existed at the 3' end of the ASP ORF and were likely determinant for the polyadenylation of the antisense transcript [4]. We next searched for the 3' end of the antisense transcript by conducting 3'RACE analyses, again using RNA from pNL4.3ΔNarI-transfected 293T cells. Through this approach, forward primers were initially designed 500 bp upstream of the ASP stop codon in the antisense strand. However, no signals reminiscent of the presence of a polyA tail being added to the antisense transcript at proximity to this region were detected (data not shown). We thus searched for a potentially new polyA signal and found an AATAAA consensus sequence at position 4908 of the pNL4.3 molecular clone (sense strand positioning) in the complementary sequence corresponding to the *pol *gene (Figure 5B). Comparison of this polyA consensus sequence among different HIV-1 strains revealed a high degree of conservation and further demonstrated its close localisation to a downstream GT-rich sequence, another marker for polyA addition [22] (Figure 5C). We thus repeated our 3' RACE analyses using the RNA sample from the same transfected 293T cells and used a forward primer at a distance closer to this potential polyA signal. After amplification, a specific signal was detected with a size expected for a polyA tail being present near the polyA signal (Figure 5A). Cloning and sequencing of the 3'RACE amplified products further confirmed that the polyA signal was next to the position of the added polyA tail (at a 19 nucleotide distance) (Figure 5C).

These results hence confirmed that the antisense transcript was polyadenylated and a newly identified and well conserved polyA signal located at 2.4 kb distance from the ASP stop codon was likely essential for the addition of the polyA tail.

### Modulation of HIV-1 antisense transcription using an antisense luciferase-expressing proviral DNA

**Figure 6:**
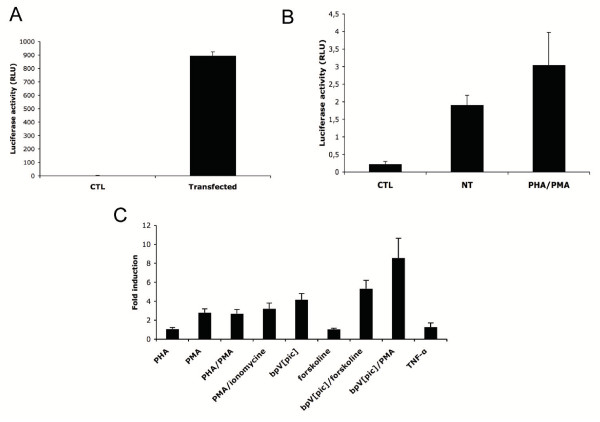
Generation of a proviral DNA construct expressing the antisense directed luciferase gene expression. (***A***) 293T cells were transfected with 600 ng pNL4.3AsLucR-E- and lysed 48 h post-transfection. CTL represents untransfected cells. (***B-C***) Jurkat cells were transfected with 15 μg pNL4.3AsLucR-E- (***B***) or pNL4.3ΔBstAsLucR-E- (***C***) and stimulated with different activators for 8 h. or left untreated. Lysed cells were then analysed for luciferase activity. Results show the mean luciferase activity values of three measured samples ± S.D (***A-B***) or fold induction compared to the unstimulated control (***C***).

Our RT-PCR approach represents an important tool in the detection of a specific signal for antisense transcription. However, the quantification of changes in antisense transcription levels in the proviral DNA context remained an essential element. Sense transcription and HIV-1 infection has been studied by several research groups using proviral DNA constructs containing the luciferase reporter gene inserted in the *nef *gene in frame with its ATG initiation codon (HXB-Luc and NL4.3LucR-E-) [23,24]. As these proviral DNA constructs produce virions upon transfection, we used the pNL4.3LucR-E- construct and removed its luciferase reporter gene to reinsert this reporter gene in the same position but in the inverse orientation. The cloning of the luciferase reporter gene in the antisense direction at this position permitted the usage of the transcription initiation sites identified above (presented in Figure 4). This vector, termed pNL4.3AsLucR-E- was tested in Jurkat and 293T cells and upon transfection, luciferase activity was constantly measured in both cell lines (293T: 893.7 ± 30 RLU and Jurkat: 2.2 ± 0.3 RLU versus 0.2 RLU as a blank value) (Figure 6A–B).

As previous results had suggested that antisense transcription was positively modulated by T-cell activators [4,19,21], we next tested our antisense luciferase-expressing proviral DNA clone for its response to T-cell activators upon transfection in Jurkat cells (Figure 6B). Our initial results confirmed previous data in that NF-κB-activating agents such as PMA and PHA slightly but significantly induced luciferase activity in transfected Jurkat cells. We next tested a version of this construct from which the 5' LTR was deleted to assess if blocking of sense transcription could impact on the level of induced antisense transcription (Figure 6C). This construct termed pNL4.3ΔBstAsLucR-E- was transfected in Jurkat cells, which were subsequently treated with a wider range of T-cell activators. Measurement of luciferase activity from these transfected cells indeed revealed that the 5'LTR-deleted version was more potent in its response, especially when comparing the responses toward PHA and PMA activation.

These results hence demonstrated that quantification of luciferase activity can be achieved using our pNL4.3AsLucR-E- construct and that, in the context of this proviral DNA, we were able to confirm induction of antisense transcription by T-cell activators. Furthermore, responses toward these T-cell activators seemed to be modulated by sense transcription.

### Upregulation of antisense transcription by the viral Tat protein

**Figure 7:**
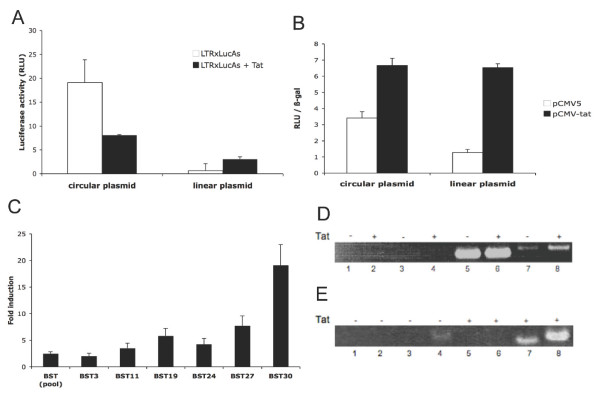
Modulatory effect of Tat on antisense transcription. (***A***) Jurkat cells were transfected with 10 μg of circular or BamHI-digested pAsLTRXLuc plasmid in combination with 5 μg of pCMV-tat or empty pCMV vector. (***B***) 293T cells were transfected with 400 ng of circular or BstZ17I-digested pNL4.3ΔBstAsLucR-E-, 200 ng of pActin-LacZ and 200 ng of pCMV-tat or empty vector. (***C***) 293T cell clones and a pooled population stably transfected with pNL4.3ΔBstAsLucR-E- were transfected with 200 ng pCMV-tat or empty vector. Results are presented as fold induction compared to empty vector. (***D***) A pool of 293T cells stably transfected for pNL4.3ΔNar1 was transfected with 5 μg of pCMV-tat or the empty pCMV5 vector. cDNA synthesis was performed with random primers. PCR amplifications were performed to detect the presence of the HIV antisense transcript (lane 3-4-7-8) using 24-6/25-3 primers. β-actin amplification was performed as control (lane 1-2-5-6). Lanes 1, 2, 3 and 4 represent control for DNA contamination to which RNA was directly added for PCR amplification. (***E***) RNA from the transfected 293T cells described in ***D ***were also used for amplification of antisense transcripts from 24-6F-synthesized cDNA using 30-20 (anchor) primer in combination with 25-3 (lane 1, 3, 5 and 7) or 26-5 (lanes 2, 4, 6 and 8) primers. Samples were tested for cDNA cleanup efficiency (lanes 1, 2, 5 and 6). Tat expression in transfected cells is indicated above the gel for both latter panels. Luciferase activities in ***A***, ***B ***and ***C ***represent the mean value of three measured samples ± S.D.

Previous studies from other groups had hinted on the possible adversary effect of Tat expression on antisense transcription, although these data might have been artefactual [4,19,21]. We thus readdressed these findings to provide a clearer role of Tat on antisense transcription using our antisense luciferase-expressing NL4.3 clone. As an initial step, we first looked at Tat modulation on a construct containing a complete LTR cloned upstream and in the inverse orientation of a luciferase reporter gene. Co-transfection experiments with this construct and a Tat expression vector (or the empty vector) along with the β-gal expression vector in the Jurkat cell line were performed and normalized luciferase activity was subsequently measured. As demonstrated in Figure 7A, Tat expression importantly reduced luciferase activity as previously shown. It is possible that Tat might importantly induce TAR-dependent sense transcription from the 3'LTR (and thus in the inverse orientation from the luciferase gene), which could lead to interference on antisense transcription. To evaluate this possibility, the antisense pLTRXLuc construct was linearized before transfection and then evaluated for its Tat response. As shown in Figure 7A, luciferase activity in this construct was now modulated positively by the viral Tat protein, although linearization led to an important reduction in basal antisense transcription. These results indeed suggested that Tat was rather a positive modulator of antisense transcription.

We next looked to confirm these data in the context of our luciferase-expressing proviral DNA. In order to determine the basal level of antisense transcription in the absence of Tat protein, we decided to use the 5'LTR-deleted pNL4.3ΔBstAsLucR-E-, which cannot produce Tat. The luciferase signal from this vector was further compared between a transfected circular form versus its linear form following digestion in the *gag *gene (thereby further eliminating possible interference from a full-circle sense transcript initiated from the 3'LTR). Upon co-transfection of pActin-LacZ, pNL4.3ΔBstAsLucR-E- and pCMV-tat (or the empty control vector) in 293T cells, normalized luciferase activity was determined. In Figure 7B, results of this transfection experiment are presented and indeed argue for a positive Tat-dependent modulation of antisense transcription in the context of the linearized proviral DNA, whereas a more reduced positive effect of Tat on antisense transcription was noted with the circular form of the plasmid. We then prepared stable 293T cell clones by transfection of linearized pNL4.3AsLucR-E (similarly digested) along with pCMV-Hyg and after selection, pooled clones were co-transfected with pActin-LacZ and pCMV-tat (or the empty control vector) (Figure 7C). Luciferase read-outs confirmed that our proviral DNA construct was still responsive to Tat expression in that Tat could upregulate luciferase gene expression in the antisense strand in the chromatin context by more 2.5 folds. When isolated clones were similarly transfected, all clones responded positively to Tat expression although at different levels (Figure 7C). Fold induction were observed to range between 2 to 17 folds in their response to Tat expression.

We also tested if Tat modulation of antisense transcription could be demonstrated at the RNA level. We initially transfected linearized pNL4.3ΔNarI in 239T cells and selected for stable transfectants, from which no HIV-1 sense expression should be detected. The resulting selected pool was then transfected with pCMV-tat (or the control vector) and pActin-LacZ. Cells transfected with comparable efficiency (as determined by β-gal activity) were then analysed by RT-PCR. In the absence of 5'LTR and 3'LTR sense transcription, the RT reaction was initially conducted with random primers (Figure 7D). PCR signals demonstrated that Tat expression in the context of this deleted proviral DNA construct was positively affecting antisense transcription (compare intensities between lanes 7 and 8). The actin signal was identical in both transfected cells (lanes 5 and 6) while DNA contamination controls presented no signals (lanes 1 to 4). To ascertain of the antisense strand specificity of the signal, cDNAs were also synthesized from this RNA with the 24-6F RT primer and subsequently analysed for antisense transcription using the protocol described in Figure 3 and the combination of two different PCR primer sets. As presented in panel E, both primer sets indicated an important increase in the PCR signal attributed to antisense transcription upon Tat expression (compare lanes 7 and 8 versus 3 and 4, respectively). The presence of the 24-6F primer in the PCR reaction was again controlled for in lanes 1–2 and 5–6, which showed no clear signal indicative of this primer contamination in the PCR reaction.

The body of results presented above illustrates that the HIV-1 Tat protein acts positively on antisense transcription in the context of a 5'LTR-deleted proviral clone.

### Detection of luciferase activity following infection of cells by antisense luciferase-expressing virions

**Figure 8:**
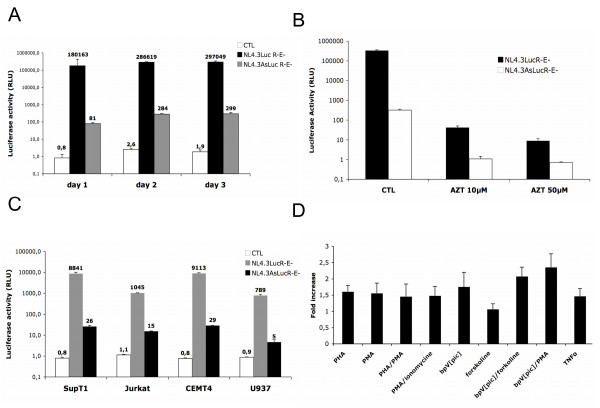
Expression of HIV-1 antisense transcript during viral infection. (***A***) 293T cells were infected with 35 ng of NL4.3LucR-E- or NL4.3AsLucR-E- virions pseudotyped with Vesicular Stomatitis Virus envelope (VSVg) and were lysed at different time points post-infection and tested for luciferase activity. In (***B***), 10 μM or 50 μM of AZT was added to the culture media 1 h before infection of 293T cells and luciferase activity was determined after 48 hr. (***C***) SupT1, Jurkat, CEMT4 and U937 cells were infected with NL4.3LucR-E- or VSVg-pseudotyped NL4.3AsLucR-E- virions at a ratio of 1 ng/1 × 10^5 ^cells. Cells were lysed 48 hr post-infection and tested for luciferase activity. (***D***) Forty-eight hr post-infection, Jurkat cells were seeded at a concentration of 1 × 10^6 ^cells/ml and stimulated with various cell activators for 8 h. Luciferase activities represent the mean value of three measured samples ± S.D. Luciferase activity in panels ***A***, ***B ***and ***C ***are presented on a logarithmic scale. Mean values of luciferase activity are indicated in panels ***A ***and ***C ***above each column.

Given that the pNL4.3AsLucR-E- vector was constructed by keeping the structure of the parental vector intact, we were thus confident that this new vector should allow us to produce virions, which could be pseudotyped and used to study antisense transcription during infection. Hence, pNL4.3AsLucR-E- and the parental pNL4.3LucR-E- were separately co-transfected with a VSV-G expression vector in 293T cells. Harvested supernatants indicated that high levels of p24 capsid proteins were detectable. Subsequently, an identical quantity of both pseudotyped virions (p24 levels) was used to infect 293T cells (Figure 8A–B). Luciferase activity was detected for both types of virions above levels measured in non-infected cells (luciferase activity is indicated above each column). Interestingly, and as expected, luciferase activity was importantly lower in 293T cells infected with NL4.3AsLucR-E virions as compared to cells infected with NL4.3LucR-E- virions. However, it was noted that for both cell lines, a continuous increase in luciferase activity was apparent for both viruses between day 1 and 2 and levelled off at day 3. Further experiments using AZT revealed the specificity of the signal in that pre-treatment of 293T cells with AZT importantly reduced the luciferase signal obtained from infection of either virus (Figure 8B). Additional infection experiments with NL4.3AsLucR-E- virions were equally conducted in other cell lines, which included T-cell lines CEMT4, SupT1 and Jurkat and the monocytic U937 cell line. These infection experiments demonstrated that, albeit at a lower extent, antisense luciferase reporter gene expression was detected in all tested cell lines above levels from uninfected cells, although important differences with NL4.3LucR-E-infected cells remained (Figure 8C). Jurkat cells infected with NL4.3AsLucR-E- virions were also tested for several T-cell activators and, as demonstrated through our transfection experiments (Figure 6), induction of luciferase expression by these agents was demonstrated, being again optimal with the bpV [pic]/PMA combination (Figures 8D).

These results thus indicated that our antisense luciferase-encoding proviral DNA can produce virions allowing quantification of antisense expression by luciferase activity in several cell types and at different time points post-infection.

## Discussion

Antisense transcription in retroviruses has remained controversial for several years. We and others have recently demonstrated that this pattern of transcription was existent in HTLV-I and further experiments have convincingly shown its coding potential and the important role played by the HBZ protein [2,5-15]. For HIV-1, antisense transcription has been poorly studied and these studies have not convinced the HIV-1 research community over the existence of both antisense transcripts and the ASP protein [4,16,18,19]. In this study, our goal was to readdress antisense transcription in HIV-1. Our results demonstrate for this first time that indeed antisense transcription does exist in HIV-1, likely involves a newly described antisense polyA consensus sequence and is positively modulated by T-cell activators and the viral Tat protein. We further present data on a new proviral DNA construct, which should allow us to quantify antisense expression in different cell types and under different conditions.

We have first set out to detect the antisense transcript in a specific fashion. Although previous studies had presented evidence based on RT-PCR analyses supporting its existence [4,18], endogenous RT priming has been a major drawback in fully acknowledging the existence of antisense transcription in HIV-1. Our first strategy to avoid endogenous RT priming was to study antisense transcription in the context where sense transcription was greatly reduced, as these transcripts are likely the major source of non-specificity masking antisense transcript-specific signals. J1.1 cells and the pNL4.3ΔNarI proviral DNA have allowed us to achieve this goal and to detect antisense transcription with a limited presence of endogenous RT priming. Indeed, an important signal of the expected size was obtained in both conditions and for pNL4.3, the presence of the 5'LTR was, as expected, resulting in endogenous RT priming. Using a more optimized RT-PCR approach, we have been successful in detecting an antisense RNA-derived PCR signal in cell lines in which sense transcription was more active. Our approach uses mRNA to avoid contaminating DNA (also in small fragments) and, through the use of a semi-complementary RT primer, strongly favours amplification of cDNAs synthesized from this primer and not from contaminating small RNA fragments. Such a comparable approach has been previously used successfully for the detection of antisense transcription in the Hepatitis C Virus [25]. An interesting observation from our data is that U937-infected cells generated a strong signal. Whether this is only specific to the cell line or representative of infected monocytic cells remain to be determined.

Upon characterization of the antisense transcript, we have demonstrated that multiple transcription initiation sites existed for antisense transcripts. It is however worth noting that these initiation sites mainly occur near the 5' border of the 3' LTR, which differs from what has been demonstrated for HTLV-I. However, one transcription initiation site agrees with the results obtained by Peeters *et al. *(1996) [19], while our current data do not support results from other groups including a recent study [4,20]. Protocols and tested cell lines might explain these discrepancies and further experiments will be needed to confirm our data on the transcription initiation sites. However, we have mapped several transcription initiation sites in this region and have confirmed these data in several experiments using a near full length proviral DNA construct (i.e. pNL4.3ΔNarI).

In our study, unlike HTLV-I antisense transcripts, no clones from our 5' RACE analyses indicated the presence of splicing events occurring in the 5' end of the antisense transcript. In addition, numerous RT-PCR experiments with primers designed from proviral DNA spanning the ASP and 3'LTR region have been undertaken and none of these experiments conducted in 293T cells transfected with different wild-type or deleted proviral DNA or HIV-1 infected cell lines has permitted to provide evidence for the existence of splicing at the 5' end of the antisense RNA. Hence, these results might justify the closer positioning of the transcription initiation sites to the ATG initiation codon of the potential ASP ORF (as compared to HTLV-I HBZ initiation codon) in order to permit sufficient translation efficiency.

Our characterization of the transcript has also permitted the identification of a new HIV-1 polyA signal. This signal is different from the previously suggested polyA signal in the study of Michael *et al. *(1994) [4]. However, we have confirmed the potential usage of our polyA signal by 3' RACE analysis. In addition, this sequence is conserved among different strains and obeys to the consensus sequence. Furthermore, a GT rich sequence is present in the neighbouring DNA region, again a potent marker for positioning of polyA tail addition in the transcript. We are thus confident that this is the polyA signal used for antisense transcription termination. The 2.4 kb length of the presumed 3'UTR suggest that this region of the transcript might play a role in the stability of the transcript, although it still remains to be determined if splicing events might be occurring at this 3' end. By Northern blot analysis, we have not been able to detect the expected 4.1 kb size of the antisense transcript in HIV-1-infected cell lines or 293T cells transfected with proviral DNA. This is likely reminiscent of low levels of the antisense transcript, which is well illustrated in our infection experiments conducted with NL4.3LucR-E- and NL4.3AsLucR-E virions. Indeed, levels of antisense transcript in these experiments are 30 to 1000 folds lower than that of sense transcripts. However, based on our 5' and 3' analyses, the Northern blot signals initially attributed to antisense transcription presented by Bukrinsky and Etkin (1990) [17] do not correspond to the expected size and furthermore these results had not subsequently been confirmed by other groups.

An important contribution from our present study is the generation of our pNL4.3AsLucR-E- construct. By transfection experiments, we have indeed demonstrated that luciferase activity was being detected and that this vector permitted the production of infectious pseudotyped virions, which produced a luciferase signal for at least three days. The construct also allowed us to confirm previous results from others in that T-cell activators could upregulate antisense transcription in both transfection and infection experiments. As opposed to these studies, we have not seen any positive effect of TNFα on luciferase activity, although other T-cell lines will need to be tested. Although this might argue that NF-κB might not be important for induction of antisense transcription, other activators known to activate this transcription factors were capable of upregulating levels of luciferase activity. Transfection experiments with 3'LTR-deleted versions will be required to pinpoint the transcription factors important for this transcriptional modulation.

Our results have also surprisingly demonstrated that Tat could upregulate antisense transcription. We have multiple evidences favouring this conclusion: transient and stable transfection experiments indeed have been conducted and luciferase reporter measurement and RT-PCR analyses all lend credence to a positive modulation by Tat. Previous studies had rather suggested a negative role but as stated in one of these studies [4,19,21], these results might be consequential of TAR-dependent sense transcription being strongly induced by Tat and having an interference effect on luciferase transcript initiated from the inverted 3'LTR. We have indeed demonstrated that this was likely the case by linearizing the vector. Similar conclusions were suggested by Bentley *et al. *who have mutated the 3'LTR TATA box and then observed a low but significant induction of antisense transcription by Tat. The mechanism by which Tat could activate the antisense promoter is unknown. Tat activates the expression of several different cellular genes through different mechanisms including binding to DNA-bound transcription factors [26]. Although current model on the action of Tat through TAR binding on sense transcription favours an increase in the processivity of the RNA polII in sense transcription, it is still possible that Tat could also act upon formation of preinitiation complexes (PIC), as it has been recently readdressed [27,28]. Indeed, several reports have indicated that Tat can interact with factors present in the preinitiation complex. However it should also be kept in mind that Tat has been shown to modulate Sp1 binding [29,30]. An important Sp1 site has previously been proposed for antisense transcription [19] and could be the target of Tat-mediated upregulation of antisense transcription. More experiments will be required to determine the mechanism by which Tat activates antisense transcription. However, we are aware that the present effect of Tat on antisense transcription was demonstrated in 5' LTR-deleted proviral DNA constructs and thus this Tat-dependent modulation of antisense transcription remains to be confirmed in the context of a full-length proviral DNA (for example, using a Tat-deficient proviral clone).

Infection experiments with NL4.3 based virions expressing an antisense reporter gene have revealed that this tool could indeed be used to study antisense transcription in the context of infection. The specificity of the luciferase signal was confirmed by the addition of AZT. Interestingly, the extent of luciferase activity was lower in tested T and monocytic cells than in 293T cells for both sense and antisense transcription. Remarkably, as pointed out above, the results from our infection experiments with the two different luciferase-encoding virions revealed that antisense transcription was importantly lower than sense transcription. A similar situation also exists for antisense transcription in HTLV-I [2,12]. This low abundance might be essential to avoid potential adverse effect of antisense transcript on sense transcripts and thereby virus replication. It is likely that sense transcription, especially upon induction by Tat or T-cell stimulating agents contribute importantly in keeping antisense RNA at low levels. It might be postulated that sense transcription initiated from the 3' LTR could also compete with limited amount of transcription factors and also diminish the extent of PIC formation on the antisense strand. In this regard, part of the function of the antisense RNA might be viewed as a true regulator of viral replication by acting as an antisense as previously suggested [31]. As another possibility, the antisense transcript might have a totally different role on HIV-1 replication as recently suggested for the HTLV-I antisense transcript [13].

Another possible function of this transcript is through its encoding potential for the ASP protein. Important information is still lacking and most importantly its detection remains an important issue to solve. The nucleotidic region of this ORF is well conserved and its presumed ATG initiation is also very well preserved. HIV-1 is very heterogenous in the sequence of its different strains, and one might thus argue that the conservation of this ORF is an indication of its encoding capacity. In addition, its position is extremely similar to the HBZ ORF in HTLV-I. Preliminary data from our group has suggested that interrupting its translation by adding an in frame stop codon importantly reduces extracellular p24 levels in transfected cells, confirming previous data from Vaquero's team [16]. We are currently conducting several experiments to address issues related to this potential viral protein.

In conclusion, we have provided strong evidence for the existence of antisense transcription in HIV-1 and our data support a positive modulation of this transcript by the viral Tat protein. We have produced an important tool, which will permit to easily assess antisense transcription following infection or transfection in the context of the proviral DNA and in the chromatin environment. These data are important as they represent an important aspect of HIV-1 replication, which has been left unanswered. In addition, the potentially encoded ASP protein needs to be further studied. Understanding the function of this protein would add to the comprehension of HIV-1 pathogenesis. In addition, its suggested membrane-bound localisation would make it a target for anti-retroviral treatment and vaccine strategies.

## Methods

### Cell lines

T-cell line used in this study included Jurkat E6.1 [32], SupT1[33] and CEMT4 [34]. Tested HIV-1 infected T-cell lines were the ACH-2 [35] and J1.1 cell lines [36]. The monocytic U937 [37], OM10.1 [38] and U937HIV-1IIIB cell lines were also used in the present study, the two latter being infected by two different HIV-1 strains. All these cell lines were maintained in RPMI-1640 medium supplemented with 10% Fetal Bovine Serum (Hyclone Laboratories, Logan, UT), 2 mM glutamine, 100 U/ml penicillin G, and 100 μg/ml streptomycin. The human embryonic kidney 293T fibroblast cell line [39] was grown in similarly supplemented DMEM.

### Plasmids and antibodies

The pCMV-tat vector was provided by Dr. O. Schwartz (Unité d'Oncologie Virale, Institut Pasteur, Paris, France) and contains the immediate early enhancer/promoter region of the human cytomegalovirus placed upstream of the viral *tat *gene [40]. The control vector pCMV5 was kindly provided by Dr. É. Rassart (Université du Québec à Montréal, Montreal Canada). The pAsLTRXLuc construct was generated by excising the XhoI/HindIII 3'HIV-1 LAI LTR fragment from pLTRXLuc and cloning this LTR fragment after blunting in SmaI-digested pGL3-basic (Promega, Naepean, Canada) in the inverse orientation relative to the luciferase reporter gene. The pNL4.3 proviral DNA clone is a full-length infectious HIV-1 molecular clone provided by the NIH AIDS Research and Reference Reagent Program (Rockville, MD). The pNL4.3LucR-E- vector (containing the luciferase reporter gene and deficient for envelope synthesis) was generously provided by Dr. N. R. Landau (The Salk Institute for Biological Studies, La Jolla, CA) [41]. The pNL4.3ΔNarI construct was derived from pNL4.3 by enzymatic digestion with NarI and self-ligation, thereby deleting the 5' LTR sequence. The pNL4.3AsLucR-E- vector was constructed by cloning the XbaI (blunted)/XhoI luciferase fragment from pGL3-basic in the NotI (blunted)/XhoI digested pNL4.3LucR-E- vector after excision of the luciferase reporter gene from this vector. The 5'LTR-deleted pNL4.3ΔBstAsLucR-E- construct was derived from the pNL4.3AsLucR-E- vector by enzymatic digestion with BstZ17I and subsequent self ligation. The pHCMV-G vector expressing the broad host-range Vesicular Stomatitis Virus envelope glycoprotein G (VSV-G) has been described previously [42]. The pActin-LacZ vector contains the lacZ gene under the regulation of the actin promoter. The hybridomas producing anti-p24 (clone 31-90-25 and 183H12-5C) antibodies were obtained from the American Type Culture Collection (Manassas VA). Antibodies from these hybridomas were purified with mAbTrap protein G affinity columns according to the manufacturer's instructions (Amersham Biosciences Inc., Uppsala, Sweden).

### Transient and stable transfections

293T cells were transfected in 24-well plates with Lipofectamine 2000 (Invitrogen; Burlington, Canada) according to manufacturer's suggestions. Transfected cells were lysed 48 h. post-transfection in a lysis buffer (25 mM Tris phosphate, pH 7.8, 2 mM DTT, 1% Triton X-100, 10% glycerol) and luciferase activity was measured with the Dynex MLX microplate luminometer (MLX Dynex Technologies, Chantilly, VA) with a single injection of a luciferase buffer (20 mM tricine, 1.07 mM (MgCO_3_)4·Mg(OH)_2_·5H_2_O, 2.67 mM MgSO_4_, 0.1 mM EDTA, 220 μM Coenzyme A, 4.7 μM D-Luciferin potassium salt, 530 μM ATP, 33.3 mM DTT). Each sample was co-transfected with the β-gal-expressing vector for normalisation. The β-galactosidase activity was measured using the Galacto-Light™ kit (Applied Biosystems, Bedford, MS) according to manufacturer's suggestions. Luciferase activity is presented in Relative Light Units (RLU) and represents the calculated mean ± SD of three transfected samples normalized by the measured β-galactosidase activity. Jurkat cells were transfected by electroporation with 15 μg of DNA using the Gene Pulser Xcell system (Bio-Rad, Hercules CA) (960 μF, 250 V). Cells were resuspended at 37.5 × 10^6^/ml of complete medium, transfected in bulk and were separated at 16 h post-transfection into various treatments group at a density of 1 × 10^5 ^cells/well (100 μl) in 96-well flat-bottom plates. Cells were either left untreated or were treated with phorbol 12-myristate 13-acetate (PMA) at 20 ng/mL (Sigma-Aldrich, Oakville, Canada), phytohemagglutinin (PHA-P, Sigma-Aldrich, Oakville, Canada) at 3 μg/mL, ionomycin (Sigma-Aldrich, Oakville, Canada) at 1 μM, bpV [pic] (Alexis corp.) at 15 μM, forskolin (Sigma-Aldrich, Oakville, Canada) at 10 μM and TNFα at 20 ng/ml. Luciferase activity was monitored as described above at 8 h. post-stimulation. Stable transfection of 293T cells were performed as follow. Linearized pNL4.3ΔBstAsLuc and pNL4.3ΔNarI constructs (upon digestion with BstZ17I and ApaI, respectively) were co-transfected with each linearized plasmid with the pCMV-Hyg vector in the presence of Lipofectamine 2000 according to manufacturer's instructions. After transfection (48 hours), Hygromycin B (Sigma-Aldrich, Oakville, Canada) was added to the cells at a concentration of 200 μg/ml. Hygromycin-resistant clones were isolated and tested for luciferase activity.

### RT-PCR and 5'/3' RACE analyses

Total RNA was extracted by the Trizol reagent (Invitrogen; Burlington, Canada). PolyA+ RNA was purified from lysed cell samples using the Poly(A)Purist™ Kit (Ambion, Austin TX) and according to manufacturer's instructions. RT-PCR analyses were conducted using the EndoFree RT Kit (Ambion, Austin TX) with RT primer 24-6 (5'-TAAAACAAATTATAAACATGTGGC-3') or the RT primer 24-6F (5'-**ATCATGAAGCATCTAGATTCAAAGTGCTGC**TAAAACAAATTATAAACATGTGGC-3') (the non-complementary sequence being indicated in bold) according to manufacturer's instructions. Briefly, polyA+ RNA (250 ng) or total RNA (10 μg) were mixed with 1 μl of 10 μM of a specific RT primer. The RNA:RT primer mix was heated at 70°C for 5 min., equilibrated at 50°C for 5 min. and incubated in the presence of the reaction mix containing 1× RT buffer, 1 μl of 2.5 mM dNTPs, 10 U of SuperAsin RNAse inhibitor (Ambion) and 1 μl RT. For certain RT reactions, cDNAs were purified through a QIAquick PCR purification kit (Qiagen) before PCR amplification. Synthesized cDNA were then PCR amplified in the presence of 1.25 U Taq DNA polymerase, 1× ThermoPol buffer, 20 μM dNTPs, 1.5 μM of each primer using a Tgradient thermocycler (Biometra, Goettingen, Germany). Primers added to the PCR reactions were the reverse primer 26-6 (5'-AAAGCAATGTATGCCCCTCCCATCAG-3') and forward primers 26-5 (5'-TGCTGTTGCGCCTCAATAGCCCTCAG-3'), 25-3 (5'-ATGCCCCAGACTGTGAGTTGCAACA-3'), or the anchor primer 30-20 (5'-ATCATGAAGCATCTAGATTCAAAGTGCTGC-3') (specifically used for the RT reactions performed in the presence of the 24-6F primer). PCR conditions were as follow: a first step of denaturation at 94°C for 5 min followed by 35 cycles of denaturation (94°C for 1 min.), annealing (60°C for 1 min.) and extension (72°C for 1 min.) and a final extension at 72°C for 5 min. RT-PCR amplifications were controlled for DNA contamination (RNA samples with no RT step), for the presence of the RT floating primer (24-6F) after cDNA purification (mixing a 24-6-synthesized cDNA with an RT reaction performed with a 24-6F floating primer in non-transfected 293T RNA) and endogenous RT priming (cDNA synthesis reaction in the presence of RT with no added specific primer). Extremities of the antisense transcript (5' and 3') were analyzed from isolated total RNA with the FirstChoice RLM-RACE kit from Ambion according to the manufacturer's instructions. For the 5'RACE protocol, RNA was ligated to a supplied anchor at their 5' end and cDNAs were subsequently synthesized with random decamers. Two PCR rounds were conducted with the supplied 5'RACE outer and inner primers and HIV-1-specific primers R1 (5'-AATCACAAGTAGCAATACAGCAGCTAA-3') and R2 (5'-CAATTATTGTCTGATATAGTGCAGCAGCAGAAC-3') successively. For the 3'RACE protocol, cDNA synthesis was performed in the presence of the supplied 3'RACE adapter. PCR amplification was achieved through 3'RACE inner and outer primers and HIV-1-specific forward primer 30-10 (5'-GGCTGGGTTCGGTATTAAGGAA-3'). Amplified products were then cloned in pBlue Script KS+ (Stratagene, La Jolla CA) and sequenced.

### Production of virus stocks

Virus particles were produced by calcium phosphate transfection of virus-encoding vectors in 293T cells as previously described [43,44]. Briefly, 293T cells were plated 16 h. before transfection to reach a 50–80% confluence at the day of transfection and then transfected with 35–40 μg through the calcium phosphate protocol as previously described [44]. Pseudotyped HIV-1 particles were generated by cotransfecting pNL4.3LucR-E- (25–30 μg) and the VSV-G envelope expression vector (5 μg of pHCMV-G). Sixteen hours after transfection, cells were washed twice with PBS and incubated for 24 h. in complete DMEM culture medium. Viruses were collected at this point by filtering the culture media through a 0.22 μm pore size cellulose acetate membrane. Virus stocks were normalized for virion content using an in-house sensitive double antibody sandwich enzyme-linked immunosorbent assay (ELISA) specific for the major core viral p24 protein as previously described [44]. Virus stocks were aliquoted and frozen at -80°C for future use. All virus stocks underwent a single freeze-thaw cycle before use in infection studies.

### Infections

293T cells were plated at 7.5 × 10^4 ^cells/well in 24-well plates at 16 h before infection. Pseudotyped virions (37.5 ng p24) were added to each well and cells were lysed at different time points post-infection with 200 μl of 1× lysis buffer. Jurkat, U937, CEMT4 and SupT1 cells were seeded in the presence of virions (1 ng of p24/10000 cells) in complete medium at 1 × 10^6 ^cells/ml and incubated for 5 h. at 37°C. Cells were then resuspended at 1 × 10^6 ^cells/ml in fresh medium and lysed at 48 h. post-infection. Luciferase activity was monitored from 25 μl aliquot of each cell lysate with the Dynex MLX microplate luminometer (MLX Dynex Technologies, Chantilly, VA) upon addition of 100 μl of luciferase buffer as described above. Mean luciferase activity ± standard deviation (SD) was calculated using luciferase activities from three independent and similarly treated cell samples taken from the same experiment.

## List of abbreviations

HBZ: HTLV-I bZIP

HIV-1: human immunodeficiency virus type 1

HTLV-I: human T-cell leukemia virus type I

LTR: long terminal repeat

PBMCs: Peripheral Blood Mononuclear Cells

AZT: Azidothymidine

## Competing interests

The author(s) declare that they have no competing interests.

## Authors' contributions

SLandry developed the antisense-specific RT-PCR protocol, designed and carried out most of the RT-PCR analyses, the 5' and 3' RACE analyses, generated most of the DNA constructs and stable cell lines, performed the transfections and infections experiments, conducted the sequence alignments and drafted the manuscript. MH has performed RT-PCR and transfection experiments. BA and SLefort have conducted several transfection experiments and have constructed certain vectors. CV and JMM have helped in drafting and finalizing the manuscript and provided important input on the design of the study. BB conceived the study, participated in its coordination, and helped in drafting the manuscript and finalizing the manuscript. All authors read and approved the final manuscript.
